# Gag-specific cellular immunity determines in vitro viral inhibition and in vivo virologic control following SIV challenges of vaccinated monkeys

**DOI:** 10.1186/1742-4690-9-S2-P245

**Published:** 2012-09-13

**Authors:** KE Stephenson, H Li, BD Walker, NL Michael, DH Barouch

**Affiliations:** 1 Beth Israel Deaconess Medical Center and Harvard University, Boston, MA, USA; 2 Ragon Institute of MGH, MIT, and Harvard, Charlestown, MA, USA; 3 US Military HIV Research Program, Walter Reed Army Inst. of Research, Silver Spring, MD, USA

## Background

A vaccine for HIV-1 would ideally block HIV-1 acquisition as well as durably control viral replication in breakthrough infections. A recently published study showed that optimal SIV vaccines can reduce SIV infection risk and setpoint viral loads following SIV challenges in rhesus monkeys, and that immunization with SIV Env was required for blocking acquisition of infection (Nature 2012 482:89-93). Here we investigate whether CD8+ T lymphocytes from these vaccinated rhesus monkeys mediate viral inhibition in vitro and whether these responses predict virologic control following SIV challenge.

## Methods

PBMC from 23 monkeys that received DNA/MVA, MVA/MVA, Ad26/MVA, or MVA/Ad26 vaccines expressing SIVsmE543 Gag/Pol/Env were used in CD8+ T-cell-mediated in vitro viral inhibition assays. CD8-depleted PBMC were infected with SIVmac251, and viral inhibition was defined as the log reduction in p27 of cultures of CD8-depleted PBMC with and without CD8+ T lymphocytes. Viral inhibition was correlated with cellular immune responses and setpoint viral loads using Spearman rank-correlation tests.

## Results

In vitro CD8+ T-cell-mediated viral inhibition prior to challenge correlated with Gag-specific ELISPOT (P=.002), total and central memory CD8+ (P<.001 for both), and total and central memory CD4+ responses (P=.002 and P=.001, respectively). A trend was observed with Gag-specific effector memory CD8+ T-cell responses (P=.014; P<.006 required for significance after multiple comparison adjustments). Viral inhibition did not correlate with Pol- or Env-specific cellular immune responses. Moreover, in vitro viral inhibition prior to challenge inversely correlated with in vivo setpoint viral loads following challenge (P=.014).

## Conclusion

These data demonstrate for the first time that the in vitro viral inhibition assay following vaccination is a predictor of in vivo virologic control following infection. Furthermore, in vitro viral inhibition correlated with Gag-specific, but not Pol- or Env-specific, cellular immune responses. These data suggest the importance of including Gag in an HIV-1 vaccine in which virologic control is desired.

